# Targeting Lysophosphatidic Acid Ameliorates Dyslipidemia in Familial Hypercholesterolemia

**DOI:** 10.34133/research.0629

**Published:** 2025-02-27

**Authors:** Zhiyong Du, Yu Wang, Fan Li, Xuechun Sun, Yunhui Du, Linyi Li, Huahui Yu, Chaowei Hu, Haili Sun, Xiaoqian Gao, Lijie Han, Zihan Zhang, Jingci Xing, Luya Wang, Jianping Li, Yanwen Qin

**Affiliations:** ^1^ Beijing Anzhen Hospital, Capital Medical University, National Clinical Research Center for Cardiovascular Diseases, Beijing 100029, China.; ^2^ Beijing Institute of Heart Lung and Blood Vessel Disease, Key Laboratory of Remodeling-Related Cardiovascular Diseases, Ministry of Education, Beijing 100029, China.; ^3^ Department of Cardiology, Peking University First Hospital, Beijing 100034, China.

## Abstract

Familial hypercholesterolemia (FH) is a lipoprotein disorder characterized by elevated plasma levels of low-density lipoprotein cholesterol (LDL-C) and an increased risk of premature atherosclerotic cardiovascular disease. Recent evidences have shown that several glycerophospholipid species were markedly altered in experimental FH animals and exhibited diverse bioactivities. Nevertheless, the glycerophospholipid profiles and their associated biological implications in human FH remain largely unknown. In this study, we sought to comprehensively delineate the glycerophospholipid phenotypes in human FH and to investigate the functional roles of key FH-altered glycerophospholipid molecules on cholesterol metabolism. Targeted analysis of 328 glycerophospholipid metabolites was used to profile the differentiated alterations in patients with homozygous FH (HoFH; *n* = 181), heterozygous FH (HeFH; *n* = 452), and non-FH hypercholesterolemia (*n* = 382). Our findings revealed that the glycerophospholipid phenotypes of FH and non-FH hypercholesterolemia were dominated by a spectrum of metabolites involved in the lysophosphatidic acid (LPA) metabolism. Among the LPA features, palmitoyl-LPA (16:0) showed significant association with the clinical levels of LDL-C and total cholesterol in HoFH and HeFH populations. Using functional metabolomic strategy and murine FH model, we demonstrated that supplementation with LPA 16:0 elevated the plasma levels of LDL and free/esterified cholesterol and exacerbated the atherosclerotic lesions. Conversely, inhibition of autotaxin-mediated LPA 16:0 production significantly ameliorated dyslipidemia. Mechanistically, we uncovered that LPA 16:0 could disrupt hepatic cholesterol homeostasis by impairing cholesterol excretion and inhibiting primary bile acid synthesis. In summary, our study offers novel insights into lipid metabolism in human FH and posits that targeting LPA metabolism may represent a promising therapeutic strategy for reducing cholesterol levels in the FH population.

## Introduction

Atherosclerotic cardiovascular disease (ASCVD) is the leading cause of morbidity and mortality worldwide [1]. Numerous evidences have demonstrated the vital roles of low-density lipoprotein cholesterol (LDL-C) as the primary driving factor of atherosclerotic progression [2,3]. Highly efficacious lipid-lowering agents or therapies can lower circulating LDL-C levels that lead to a markedly decreased morbidity and mortality of ASCVD [4]. However, a substantial proportion of patients with severe hypercholesterolemia, especially individuals with familial hypercholesterolemia (FH), cannot achieve an ideal LDL-C level even after potent lipid-lowering treatment [5–7].

FH is an autosomal-dominant disorder that is typically caused by highly penetrant mutations in the genes encoding the LDL receptor (*LDLR*) [8]. Heterozygous FH (HeFH) is usually caused by a single pathogenic variant with an estimated frequency of 1 in 300 to 500 within the general population [9]. Homozygous FH (HoFH) is a rare and most severe condition that is caused by biallelic pathogenic variants with an estimated prevalence of 1 in 160,000 to 300,000 [10,11]. Patients with FH are characterized by lifelong highly elevated plasma levels of LDL-C, leading to early-onset atherosclerosis and premature coronary heart diseases [12]. In patients with HoFH or severe phenotype of HeFH, cumulative cholesterol deposits are evident in the skin, tendons (xanthomas), or around the iris (corneal arcus) [10–13].

Glycerophospholipids are key components of the cholesterol-rich lipoproteins and lipid bilayer of cells [14]. An increasing body of evidence revealed that glycerophospholipid metabolism was remarkably perturbed in dyslipidemia [15]. Several bioactive glycerophospholipids play as potent lipid mediators that may be involved in the interactions between genetic and environmental factors that increase susceptibility to atherosclerosis and its complications [16–18]. More recently, our untargeted metabolomics study showed that several glycerophospholipid metabolites were significantly altered in the blood samples of patients with HoFH and HeFH [19]. However, the comprehensive and precise glycerophospholipid landscapes of FH and non-FH hypercholesterolemic populations are still lacking.

In this study, we sought to systematically depict the plasma glycerophospholipid phenotypes and characterize the differentiated features of 1,292 individuals with and without FH, including HoFH, HeFH, non-FH hypercholesterolemia, and nondyslipidemia. We also aimed to investigate the association of the key glycerophospholipid alterations with clinical cholesterol levels and explore the potential roles of key glycerophospholipid alterations on cholesterol metabolism.

## Results

### Demographic and clinical characteristics of all participants

A total of 633 FH patients with genetically confirmed *LDLR* mutations from 2 independent FH centers (Beijing Anzhen Hospital and Peking University First Hospital) participated in this study, containing 181 patients with HoFH (male, 52.5%) and 452 subjects with HeFH (male, 51.3%). Another 112 non-FH individuals (male, 55.4%) were enrolled as a comparison group. Clinical characteristics are presented in Table S1. Patients with HoFH had the highest levels of LDL-C (14.12 ± 5.36 mM) compared with HeFH (5.31 ± 1.37 mM) and non-FH (2.21 ± 0.43 mM) individuals. Besides, historical ASCVD was recorded in 32 HoFH patients (17.6%). In the non-FH hypercholesterolemic set, a total of 382 patients (male, 51.1%) were enrolled from Beijing Anzhen Hospital (Table S2). Compared to the nondyslipidemic individuals (*n* = 165; male, 54.6%), non-FH hypercholesterolemic patients showed higher levels of LDL-C and total cholesterol (TC). No significant difference was observed in the prevalences of hypertension and diabetes mellitus in each subgroup comparison.

### Plasma glycerophospholipid phenotypes of individuals with FH and non-FH hypercholesterolemia

Using liquid chromatography–mass spectrometry (LC-MS), we profiled 328 circulating glycerophospholipid metabolites in the plasma samples of the study subjects, including 14 subclasses as follows: phosphatidylcholine (PC), ether linkage-PC (PC-O), phosphatidic acid (PA), phosphatidylethanolamine (PE), phosphatidylglycerol (PG), phosphatidylserine (PS), phosphatidylinositol (PI), lysophosphatidylcholine (LPC), O-alkyl LPC (LPC-O), lysophosphatidic acid (LPA), lysophosphatidylethanolamine (LPE), lysophosphatidylglycerol (LPG), lysophosphatidylinositol (LPI), and lysophosphatidylserine (LPS). The principal components analysis (PCA) score plot of quality control (QC) samples and the values of the coefficient of variation and relative SD for all deuterated standard substances in the QC samples are shown in Fig. S1A and B and Table S3, respectively. The results indicated that the proposed metabolomic approach was robust and reliable for further statistical analysis.

**Fig. 1.**
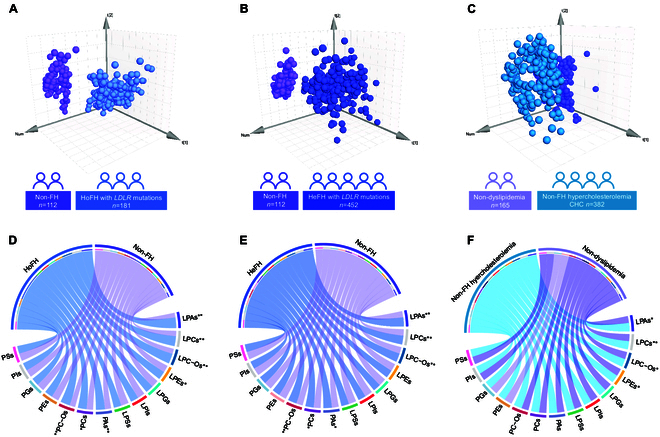
Plasma glycerophospholipid phenotypes and characteristics of patients with HoFH, HeFH, and non-FH hypercholesterolemia. (A and B) Principal components analysis (PCA) score plots based on the lipidomic datasets from HoFH, HeFH, and non-FH individuals. (C) PCA score plot based on the datasets from non-FH hypercholesterolemia and nondyslipidemia subjects. (D to F) Mean expression level-based chord diagrams of 14 glycerophospholipid subclasses between different group comparisons. ** or * indicated *P* < 0.01 or *P* < 0.05. HeFH, heterozygous familial hypercholesterolemia; HoFH, homozygous familial hypercholesterolemia; PC, phosphatidylcholine; PC-O, ether linkage-PC; LPC, lysophosphatidylcholine; LPC-O, O-alkyl LPC; PA, phosphatidic acid; LPA, lysophosphatidic acid; PE, phosphatidylethanolamine; LPE, lysophosphatidylethanolamine; PG, phosphatidylglycerol; LPG, lysophosphatidylglycerol; PS, phosphatidylserine; LPS, lysophosphatidylserine; PI, phosphatidylinositol; LPI, lysophosphatidylinositol.

Using the datasets of all detected glycerophospholipid metabolites, we subsequently used unsupervised PCA to obtain a comprehensive overview of glycerophospholipid phenotypes in different comparisons. The resultant plots indicated a distinct group separation between HoFH and non-FH groups (R2X = 0.61; Q2 = 0.52; Fig. 1A). Additionally, a clear separation between HeFH and non-FH was also achieved by PCA score plot (R2X = 0.54; Q2 = 0.46; Fig. 1B). However, the PCA score plot showed unsatisfactory classifications between non-FH hypercholesterolemia and nondyslipidemia groups (R2X = 0.38; Q2=0.25; Fig. 1C). These results suggested that the defective *LDLR* mutations might cause prominent changes in the plasma glycerophospholipid of FH patients.

### The glycerophospholipid characteristics of FH and non-FH hypercholesterolemia were dominated by abnormalities in LPA subclass

The univariate chord diagram was first employed to visualize the differences in the glycerophospholipid subclasses between different groups. The results demonstrated that patients with HoFH or HeFH showed higher levels of total PCs, PC-Os, LPCs, LPC-Os, PAs, and LPAs compared with the non-FH individuals (*P* < 0.05; Fig. 1D and E). Furthermore, our results indicated that patients with non-FH hypercholesterolemia also had higher levels of total LPAs, LPCs, and LPC-Os than the subjects with nondyslipidemia (*P* < 0.05; Fig. 1F).

**Fig. 2.**
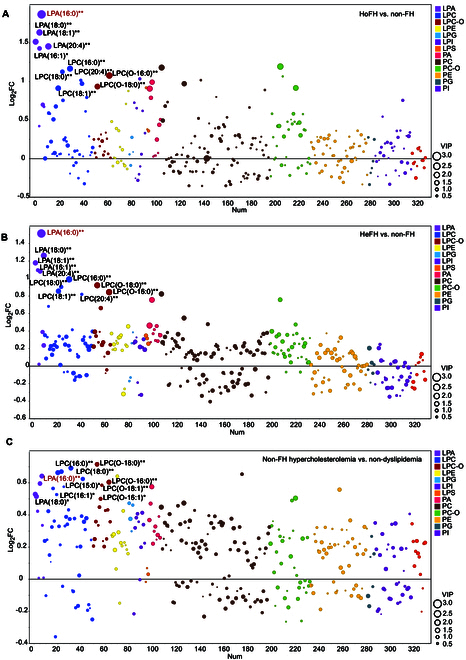
Identification of the differentiated glycerophospholipids using the multivariate and univariate statistical integration plots. (A) Integration plot depicting the differentially expressed glycerophospholipid species between HoFH and non-FH individuals based on the variable importance projection (VIP) values, fold change (FC) values, and *P* values. (B) Integration plot depicting differentially expressed glycerophospholipid species between HeFH and non-FH individuals. (C) Integration plot depicting differentially expressed glycerophospholipid species between the non-FH hypercholesterolemia and nondyslipidemia individuals. ** or * indicated univariate *P* < 0.01 or *P* < 0.05.

Based on the univariate *P* values and supervised partial least squares discriminate analysis (PLS-DA)-derived variable importance projection (VIP) values, we constructed an integrated plot to identify differentiated glycerophospholipid species between different groups. Our results identified a variety of LPAs and LPCs, and LPC-Os with higher fold change values (>2.4) and VIP values (>1.8) that were significantly elevated in HoFH patients compared to non-FH subjects (Fig. 2A), and those lysophospholipid alterations mainly consist of several long-chain fatty acyls, including palmitoyl (16:0), stearyl (18:0), palmitoleoyl (16:1), oleyl (18:1), and arachidonyl (20:4). In patients with HeFH (Fig. 2B), the HoFH-related lysophospholipid markers were also observed to be significantly elevated compared to non-FH individuals (fold change > 1.5; *P* < 0.05; VIP > 1.5). Notably, our results showed that alterations in the LPA subclass exhibited maximized differences between patients with FH and non-FH subjects. Besides, our results demonstrated that several LPCs and LPAs were also increased in the plasma of patients with non-FH hypercholesterolemia compared with nondyslipidemia (fold changes > 1.25; *P* < 0.05; VIP > 1.3). These 2 altered lysophospholipid species were found to be the major discriminated glycerophospholipid subclasses between non-FH hypercholesterolemia and nondyslipidemia populations (Fig. 2C).

**Fig. 3.**
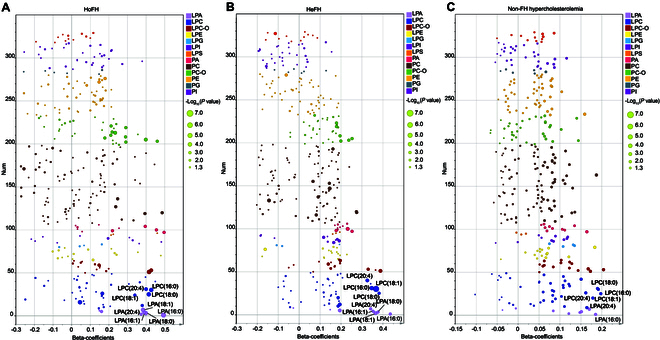
Associations between the plasma glycerophospholipids and different hypercholesterolemia conditions by using multivariate regression analyses. (A) β-Coefficient-based association plot of glycerophospholipids in the HoFH population. (B) β-Coefficient-based association plot of glycerophospholipids in HeFH subjects. (C) β-Coefficient-based association plot of glycerophospholipids in non-FH hypercholesterolemia individuals. Age, sex, LLT, ASCVD history, hypertension, and type 2 diabetes mellitus were included as adjusted covariates in all multivariate regression analyses. β-Coefficient > 0 or < 0 combined with a *P* value < 0.05 (−log_10_ *P* > 1.3) indicated a positive or negative correlation.

Considering that lipid-lowering therapy (LLT) is a potential factor that might affect the plasma lipid levels, we then employed unsupervised PCA to explore the potential effects of LLT on the glycerophospholipid profiles of different study groups. As shown in the Fig. S1C and D, the glycerophospholipid profiles did not show clustering of samples by LLT status in the PCA score plots of FH and non-FH hypercholesterolemia populations, indicating no discriminatory features due to the differences in LLT status. Furthermore, we also performed multivariate regression analyses to assess the integrated effects of several important metabolic factors (adjusted covariates included LLT, age, sex, ASCVD history, hypertension, and type 2 diabetes mellitus) on the differentiated glycerophospholipid species. As shown in the Fig. 3A to C, our results demonstrated that most of the lysophospholipid markers (LPA and LPC species) that were identified by the multivariate and univariate statistical analyses were still significantly associated with HoFH, HeFH, or non-FH hypercholesterolemia conditions after adjustments (*P* < 0.05). These results indicated that the commonly known metabolic factors showed few effects on the characteristic glycerophospholipid profiles in patients with FH or non-FH hypercholesterolemia.

### Plasma levels of palmitoyl-LPA (16:0) significantly associated with LDL-C and TC in FH population

The above results indicated that LPA was the most dominant glycerophospholipid subclass in the plasma of hypercholesterolemia population, especially in the individuals with FH. To explore the associations of LPA levels with clinical LDL-C and TC concentrations, correlation analysis was performed by using Spearman’s rank coefficients plot. As shown in Fig. S2A, the results showed that 4 LPA species (namely, 16:0, 18:0, 18:1, and 20:4) and total LPAs were positively associated with LDL-C and TC in the HoFH population, whereas palmitoyl-LPA (16:0) demonstrated the most statistically positive correlation (LDL-C, coefficient = 0.67; TC, coefficient = 0.61). In the HeFH population (Fig. S2B), total LPA and 3 LPA species (including LPA 16:0, 18:0, and 18:1) were also positively correlated with LDL-C and TC. Impressively, LPA (16:0) showed the most significant association (LDL-C, coefficient = 0.58; TC, coefficient = 0.51). However, in the non-FH hypercholesterolemia population, only LPA 16:0 was statistically correlated with LDL-C (coefficient = 0.46) and TC (coefficient = 0.44), and total LPA and other individual LPA only showed a nonstatistically positive association (Fig. S2C). Moreover, LPA 16:0 also showed independent associations with the conditions of HoFH, HeFH, or non-FH hypercholesterolemia in the multivariate regression analyses (Fig. 3). Collectively, our results demonstrated that the saturated LPA 16:0 might be a potential metabolic marker in genetic and non-genetic hypercholesterolemia populations.

### Adding LPA 16:0 to standard chow diet caused hypercholesterolemia in *LDLR^−/−^* and wild-type C57BL/6J mice

**Fig. 4.**
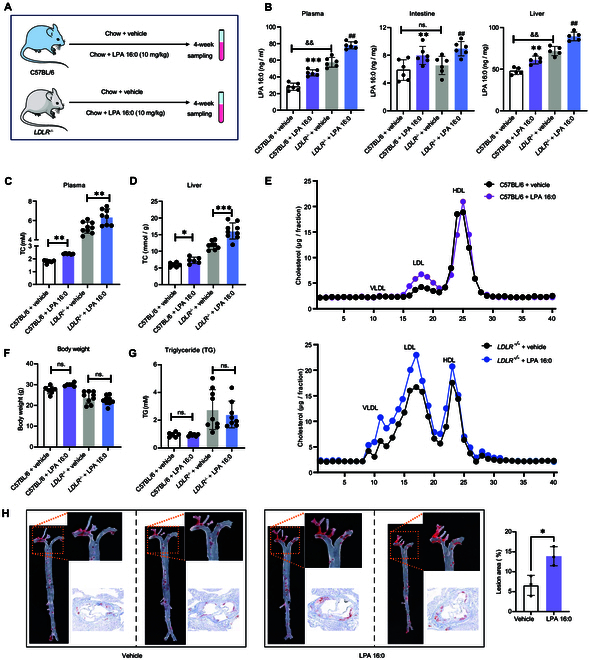
Effects of dietary LPA 16:0 supplementation on cholesterol levels and atherosclerosis in *LDLR^−/−^* mice. (A) Study design for investigating the effects of chow-containing LPA 16:0 on cholesterol levels in C57BL/6J background *LDLR^−/−^* and wild-type (WT) mice. (B) Plasma, liver, and intestine levels of LPA 16:0 after 4 consecutive weeks of chow supplementation. (C and D) Plasma and liver tissues of TC. (E) Cholesterol content in very-low-density lipoprotein (VLDL), LDL, and high-density lipoprotein (HDL) fractioned by fast protein liquid chromatography. (F) Body weight. (G) Plasma levels of triglyceride (TG). (H) Representative Oil Red O staining of the whole aorta. * and ** indicated *P* < 0.05 and *P* < 0.01, respectively.

To explore the in vivo effects of LPA 16:0 on the cholesterol levels, we fed C57BL/6J background *LDLR^−/−^* (genetic model that mimics human HoFH) and wild-type (WT) mice with chow diet containing 10 mg/kg LPA 16:0 (Fig. 4A). After 4 consecutive weeks of feeding, the in vivo concentrations of LPA 16:0 in different groups were determined by LC-MS (Fig. 4B). Compared to vehicle-treated groups, we found that the LPA 16:0 levels in the plasma, liver, and intestine were significantly increased in both *LDLR^−/−^* and WT mice after LPA 16:0 treatment. Of note, we also observed that the plasma and liver levels of LPA 16:0 in vehicle-treated *LDLR^−/−^* mice were higher than those in vehicle-treated WT mice, suggesting that *LDLR* gene deficiency could directly lead to an increase in the expression levels of LPA 16:0.

LPA 16:0 could also induce a significant elevation of TC in the plasma and liver tissue samples of *LDLR^−/−^* and WT mice (Fig. 4C and D). Furthermore, LPA 16:0-treated groups displayed markedly elevated cholesterol levels in the LDL fraction compared with vehicle-treated groups (Fig. 4E). However, the body weight and plasma levels of triglyceride (TG) did not significantly alter between LPA 16:0-treated and vehicle groups (Fig. 4F and G). In high-cholesterol diet (HCD) feeding *LDLR^−/−^* mice, supplementation with 8 consecutive weeks of LPA 16:0 (10 mg/kg) resulted in significant atherosclerotic lesions in the aorta compared with vehicle-treated mice, as evidenced by Oil Red O staining on aortic root sections (Fig. 4H).

With regard to our recent untargeted metabolomic studies in the HoFH and HeFH population, our results found that palmitic acid, the endogenous upstream or fundamental substrate for LPA 16:0, was also significantly increased in the serum samples of patients with HoFH and HeFH compared to the non-FH individuals [19]. Therefore, we also explored whether supplementation with palmitic acid (10 mg/kg; the same dose with LPA 16:0) could also alter the circulating lipid levels of *LDLR^−/−^* mice (Fig. S3A). As shown in Fig. S3B, our results indicated that the plasma and liver levels of LPA 16:0 were not significantly increased after 4 consecutive weeks of palmitic acid treatment. Compared to the vehicle-treated group, the plasma levels of TC and TG, the body weight, and the LDL fraction were not significantly altered in the palmitic acid-treated group (Fig. S3C to F). Our results showed that LPA 16:0 could elevate the plasma levels of free cholesterol and cholesterol ester (ChE; including 16:0, 16:1, 18:0, 18:1, and 18:2) in *LDLR^−/−^* mice, whereas palmitic acid could only increase the plasma levels of ChE (16:0) and showed few effects on the levels of free cholesterol and other types of ChE (Fig. S3G).

### Inhibition of autotaxin-mediated LPA 16:0 production ameliorated dyslipidemia in *LDLR^−/−^* mice

**Fig. 5.**
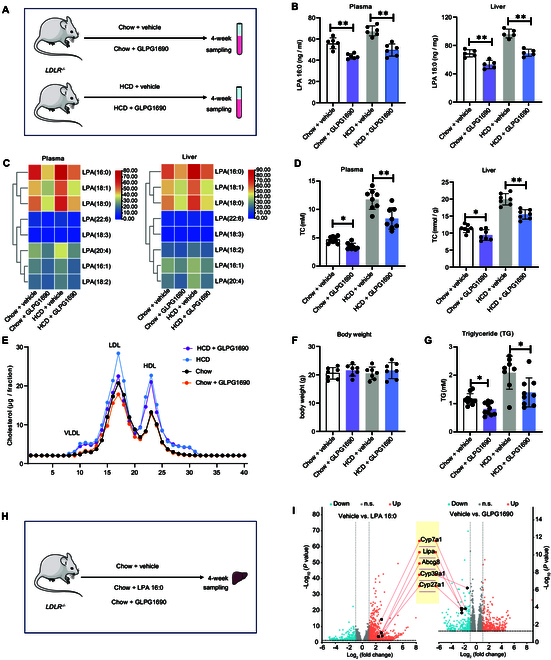
Effects of ATX inhibitor on LPA production and cholesterol metabolism in *LDLR^−/−^* mice. (A) Study design for investigating the roles of ATX inhibitor (GLPG 1690) under chow and high-cholesterol diet (HCD). (B) Plasma and liver levels of LPA 16:0 after GLPG 1690 treatment. (C) Plasma and liver levels of all detected LPA species after GLPG 1690 treatment. (D) Plasma and liver tissues of TC. (E) Cholesterol content in VLDL, LDL, and HDL fractioned by fast protein liquid chromatography. (F) Body weight. (G) Plasma levels of TG. * and ** indicated *P* < 0.05 and *P* < 0.01, respectively. (H) Study design for exploring the effects of LPA 16:0 and ATX inhibitor on hepatic cholesterol metabolism. (I) Integrated volcano plot of transcriptomic data in pairwise comparisons of vehicle versus LPA 16:0 and vehicle versus GLPG 1690. The significant threshold for the one-dimensional differences was *P* < 0.05, fold change > 1.5.

The above results demonstrated that *LDLR* gene deficiency in humans and mice exhibited a remarkable increase in the plasma levels of LPA 16:0, and adding LPA 16:0 to mouse chow could cause high cholesterol in *LDLR^−/−^* mice. Therefore, we hypothesized that inhibiting endogenous LPA 16:0 production might reduce *LDLR* gene deficiency-induced high cholesterol and LDL-C levels. LPAs are mainly derived from LPCs by the secreted enzyme autotaxin (ATX) through its lysophospholipase D activity [20]. To investigate whether depletion of LPA 16:0 could decrease cholesterol, chow- and HCD-feeding *LDLR^−/−^* mice were treated with ATX inhibitor (namely, GLPG 1690) for 4 weeks (Fig. 5A). Compared with the vehicle-treated group (Fig. 5B and C), GLPG 1690-treated group displayed lower plasma and liver levels of LPA 16:0 and several other LPAs (18:0; 18:1; 18:2; 16:1; 20:4). As we expected, GLPG 1690 could also reduce the plasma and hepatic levels of TC (Fig. 5D), and decrease the cholesterol levels in the LDL fraction (Fig. 5E). No statistical differences were observed in body weight between treated and nontreated groups (Fig. 5F). Interestingly, we also observed that GLPG 1690 treatment could significantly decrease the plasma levels of TG in *LDLR^−/−^* mice (Fig. 5G).

### ATX–LPA 16:0 axis showed potential roles on regulating cholesterol excretion and bile acid synthesis in *LDLR^−/−^* mice

**Fig. 6.**
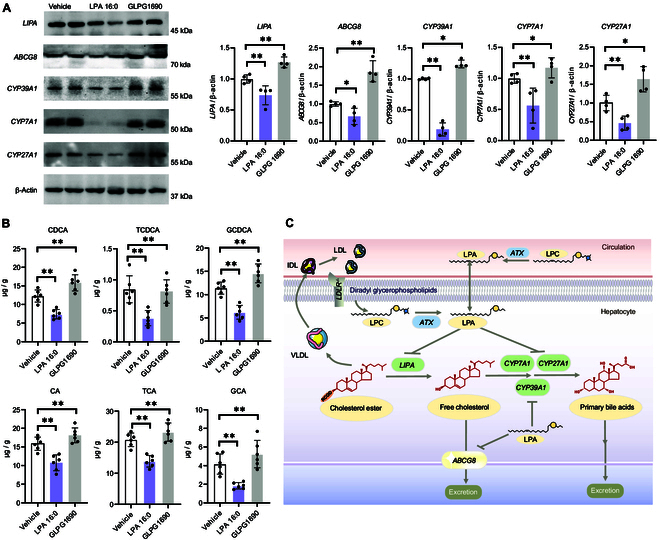
Effects of LPA 16:0 and ATX inhibitor on hepatic cholesterol excretion and bile acid synthesis in *LDLR^−/−^* mice. (A) Western blot evaluation and relative quantification of the key cholesterol metabolism-related mRNAs identified in the transcriptomic analysis. (B) Liver levels of primary bile acids between different groups. (C) Mechanism diagram of ATX–LPA 16:0 on regulating cholesterol homeostasis. * and ** indicated *P* < 0.05 and *P* < 0.01, respectively. CA, cholic acid; CDCA, chenodeoxycholic acid; GCA, glycocholic acid; TCA, taurocholic acid; GCDCA, glycochenodeoxycholic acid; TCDCA, taurochenodeoxycholic acid.

We next aimed to investigate the action mechanism of ATX–LPA 16:0 axis on regulating the cholesterol levels in *LDLR^−/−^* mice. Transcriptomics was first performed by using the liver tissues collected from LPA 16:0-treated mice, ATX inhibitor-treated mice, and vehicle-treated mice (Fig. 5H). In the volcano plots (Fig. 5I), we found that the mRNA levels of 2 genes involved in ChE hydrolysis (namely, *LIPA*) and cholesterol excretion (*ABCG8*) were differentially expressed. In the Western blot analysis, the protein levels of *LIPA* and *ABCG8* were also found to be decreased in the LPA 16:0-feeding group, and were significantly reversed after the GLPG 1690 treatment (all *P* values < 0.05; Fig. 6A). We also observed that the mice in the LPA 16:0-feeding group exhibited decreased fecal levels of free cholesterol. Conversely, GLPG 1690 treatment could significantly increase the fecal levels of free cholesterol in the *LDLR^−/−^* mice (Fig. S4A), suggesting that ATX-mediated LPA 16:0 production might play an important role in regulating hepatic cholesterol excretion.

Conversion of cholesterol to primary bile acids by hepatic CYP450 enzymes is an important process for maintaining cholesterol homeostasis. Notably, we observed that the mRNA and protein levels of 3 bile acid synthesis-associated genes (including *CYP27A1*, *CYP7A1*, and *CYP39A1*) were significantly reduced in the liver tissues of LPA 16:0-treated group (Figs. 5I and 6A). In line with this, we found that LPA 16:0 could significantly decrease the hepatic levels of 6 primary bile acids (Fig. 6B). Additionally, our results indicated that LPA 16:0 caused a remarkable decrease in the fecal levels of primary bile acids (Fig. S4B and C). On the contrary, ATX inhibitor could increase the 3 CYP enzymes levels and elevate the bile acid productions in the liver and feces of *LDLR^−/−^* mice. These results demonstrated that the activation of ATX–LPA 16:0 axis might perturb the hepatic cholesterol homeostasis by impairing primary bile acid synthesis and excretion (Fig. 6C).

## Discussion

To our knowledge, this study represents the first comprehensive characterization of glycerophospholipid metabolism phenotypes in patients with FH and non-FH hypercholesterolemia. The main findings of our study are as follows: First, patients with FH and non-FH hypercholesterolemia exhibited remarkable alterations in a variety of lysophospholipids. Second, LPA species, particularly LPA 16:0, showed a significant correlation with circulating cholesterol levels in patients with HoFH and HeFH. Third, our study demonstrated that inhibiting ATX-mediated LPA 16:0 production could markedly reduce circulating cholesterol levels by modulating hepatic cholesterol excretion and primary bile acid synthesis in a genetic mouse model that mimics human FH. These findings provide potential intervention targets for ameliorating severe hypercholesterolemia in the FH population.

In general, glycerophospholipids can be subdivided into diacyl (also referred to as phospholipids) and mono (lysophospholipids) classes. Phospholipids consist of a glycerol backbone with a polar head group at the *sn*-3 position and 2 fatty acid chains esterified at the *sn*-1 and *sn*-2 positions [21]. Phospholipids are the fundamental biomolecules of cellular plasma membrane leaflets and organelles that perform important membranes functions, such as compartmentalization of cells and separation of cells from the external environment [22,23]. Unlike phospholipids, lysophospholipids contain only one fatty acyl chain at the *sn*-1 (alkylacyl) position or *sn*-2 position (alkenylacyl) of the glycerol backbone [21]. Lysophospholipids are highly present in blood and exist in free form or bound to transport proteins such as lipoproteins and albumin [24]. In spite of their simple chemical structure, lysophospholipids have been found to be functional metabolites, serving as endogenous signaling mediators or enzyme activators [25,26].

LPC is the most abundant lysophospholipids of atherogenic lipoproteins (including LDL and ox-LDL) [27]. It has been reported that the LPC species can be derived from polar surface PC of LDL by the phospholipase A2 or produced during LDL oxidation through enzymatic hydrolysis of PC by the LDL-associated platelet-activating factor acetylhydrolase [28,29]. In our study, a variety of LPC species were found to be increased in the plasma of FH compared to those in non-FH. In addition, their levels were also found to be elevated in the plasma of non-FH hypercholesterolemia compared to those in nondyslipidemia. Understandably, the elevated circulating levels of LPC might be a consequence of higher LDL levels in patients with hypercholesterolemia. In previous in vitro and in vivo studies, LPC has been shown to possess pro-atherogenic and proinflammatory effects [30,31], including stimulation of macrophage activation, up-regulation of adhesive molecules, inhibition of endothelial relaxation, and chemotaxis of monocytes and T lymphocytes.

Impressively, our results demonstrated that LPAs were identified as the foremost lysophospholipid alterations in patients with FH, especially in homozygotes. In a murine HoFH model caused by genetic *LDLR* defect, LPA species were also found to be significantly increased in the plasma, which was in line with previous animal studies [32,33]. Besides, our results also demonstrated that the LPA concentrations were also increased in the liver tissues of *LDLR^−/−^* mice compared to those of WT mice. Numerous evidence has demonstrated that LPA has a plethora of biological responses, contributing to progressions of inflammation, thrombosis, and atherosclerosis [34,35]. The present study also indicated that the addition of LPA 16:0 could accelerate the processes of atherosclerotic lesions in HoFH model mice.

In this study, LPA 16:0 showed a significantly positive correlation with the levels of LDL and TC in the HoFH, HeFH, and non-FH hypercholesterolemia population. However, few studies have investigated the biological relationships between LPA 16:0 and cholesterol. Here, our results indicated that adding this hypercholesterolemia-associated lysophospholipid marker could have direct effects on increasing plasma and liver cholesterol levels in both *LDLR^−/−^* and WT mice, suggesting an important cholesterol-raising role of LPA 16:0.

ATX is the key enzyme that extracellularly hydrolyzes LPC and other lysophospholipids into LPA. It has been documented that ATX in endothelial cells enhances atherosclerosis through the production of LPA 16:0 and LPA 18:0, and knockdown of ATX in endothelial cells reduces lesion macrophage accumulation, thereby reducing atherosclerosis in *APOE^−/−^* mice [36]. Furthermore, previous studies also demonstrated that enterocyte-specific deletion of ATX could decrease plasma cholesterol, inhibit systemic inflammation, and reduce atherosclerosis in *LDLR^−/−^* mice [37]. Notably, the present work also revealed that the use of the ATX inhibitor (GLPG 1690) could significantly reduce LPA 16:0 and cholesterol levels in *LDLR^−/−^* mice. The in-depth studies on exploring the effects of ATX inhibitors on atherosclerosis progression in *LDLR^−/−^* mice are warranted. Moreover, studies on investigating the long-term effects and safety assessments of ATX inhibition are also worthwhile. Additionally, further studies designed to investigate the genetic associations of ATX and clinical phenotypes in the FH population may offer great promise for exploring additional intervention strategies to lower cholesterol and combat atherosclerosis progression.

In subsequent mechanistic studies, our results suggested that LPA 16:0 might regulate the hepatic cholesterol homeostasis via 2 cholesterol metabolism signaling pathways. *LIPA* is an important lipid hydrolase that hydrolyzes cholesteryl esters into free cholesterol [38], and *ABCG8* plays a key role in promoting the excretion of free cholesterol in hepatocytes [39,40]. Our result indicated that adding LPA 16:0 could inhibit the expressions of LIPA and *ABCG8*, which might contribute to the accumulation of ChEs, the commonly known substrates for very-low-density lipoprotein (VLDL) synthesis. Conversion of cholesterol to bile acids is another critical process for maintaining cholesterol homeostasis and preventing accumulation of free cholesterol in the liver [41,42]. Interestingly, our results found that LPA 16:0 could reduce primary bile acid conversion from cholesterol by inhibiting 3 hepatic CYP enzymes in *LDLR^−/−^* mice. Conversely, inhibition of ATX-mediated LPA 16:0 production could significantly reverse the expression levels of these altered cholesterol metabolism-associated molecules in *LDLR^−/−^* mice.

In summary, our study systematically characterized the plasma glycerophospholipid profiles in a large set of patients with HoFH, HeFH, and non-FH hypercholesterolemia. Our results revealed that circulating LPAs, particularly LPA 16:0, were significantly elevated in HoFH and HeFH individuals. These LPA species exhibited strong correlations with clinical cholesterol levels and demonstrated a potential role in disrupting hepatic cholesterol homeostasis. Our findings provide novel insights into the perturbations of lipid metabolism in FH and suggest a potential therapeutic strategy for targeting LPA metabolism to mitigate hypercholesterolemia in this population.

## Materials and Methods

### Study population

All FH patients were enrolled from the Familial Hypercholesterolemia Families Cohort (FHFC) affiliated to Beijing Anzhen Hospital and Peking University First Hospital between 2018 and 2023. This study complies with the Declaration of Helsinki and was approved by the Ethics Committee of Beijing Anzhen Hospital of the Capital University of Medical Sciences and Peking University First Hospital. Ethics approval was obtained from the Ethics Committee of Beijing Anzhen Hospital of the Capital University of Medical Sciences (2017035; ZD2024002) and Peking University First Hospital (2022143-005). Verbal and written consent was obtained from all subjects.

All FH patients were genetically confirmed with 2 (HoFH) alleles or 1 mutant (HeFH) allele in genes encoding *LDLR*. Non-FH was defined as subjects with lower levels of untreated LDL-C than 4.7 mM and without FH-related mutations in gene encoding *LDLR*, apolipoprotein B, proprotein convertase subtilisin/kexin type 9, or *LDLR* adaptor protein 1. ASCVD history for the subjects at inclusion was defined as a composite of myocardial infarction, coronary and carotid revascularization, and ischemic or atherothrombotic stroke. Non-FH hypercholesterolemia and nondyslipidemia individuals were enrolled from Beijing Anzhen Hospital between 2018 and 2022. Non-FH hypercholesterolemia was defined as patients with LDL-C levels ranging from 3.65 to 4.7 mM and without FH-related gene mutations in genetic sequencing. Nondyslipidemia was defined as individuals without clinical diagnosis of hypercholesterolemia, hyperphytosterolemia, and hypertriglyceridemia or history of lipid-lowering medications. Exclusion criteria for all the study subjects were as follows: current or historical diseases or conditions of respiratory diseases, serious digestive diseases, infectious diseases, chronic kidney diseases, pregnancy, and malignancy.

### Routine blood lipid measurements

Blood samples were collected from the antecubital vein after an overnight fasting state of 10 to 12 h and then stored at −80 °C until analysis. The levels of LDL-C, TC, TG, and high-density lipoprotein (HDL) were determined using an automatic biochemistry analyzer (Beckman AU 5400, Brea, USA).

### Plasma glycerophospholipid extraction and analyses

For human study, a total of 100 μl of plasma was first mixed with 200 μl of methanol containing 10 μl of internal deuterated phospholipid standards (LPA 16:0-*d*_9_, PA 15:0/18:1-*d*_7_, LPC 14:0-*d*_7_, PC 16:0-*d*_3_, LPE 16:0-*d*_9_, PE 17:0/18:1-*d*_5_, PC 16:0/18:2-*d*_5_, LPI 19:0-*d*_5_, PI 17:0/16:1-*d*_5_, LPS 19:0-*d*_5_, PS 17:0/18:1-*d*_5_, LPG 19:0-*d*_5_, PG 17:0-16:1-*d*_5_), and then 800 μl of methyl tert-butyl ether (MTBE) was added. The mixture was adequately vortexed, sonicated for 30 min at 4 °C, and then kept for 20 min. After that, 200 μl of LC-MS-grade deionized water was added, and the mixture was vortexed and centrifuged at 14,000 rpm for 15 min at 4 °C. The upper organic solvent layer was obtained and dried under nitrogen. Then, the samples were redissolved in 200 μl of isopropanol/acetonitrile (9:1, v/v) for further LC-MS analysis. For animal study, a total of 100 μl of plasma and 50 mg of liver were used, and glycerophospholipid extraction was the same as that in human study.

The LC-MS analysis was performed on an ultrahigh-performance liquid chromatography (UHPLC) system (LC-30AD, Shimadzu) coupled with QTRAP MS (6500+, Sciex) platform at Novogene Co. Ltd. The analytes were separated on a C18 column (Phenomenex, Kinetex C18, 2.1 × 100 mm, 2.6 μm). Column temperature was set at 45 °C. Mobile phase A: 70% acetonitrile + 30% H_2_O + 5 mM ammonium acetate, mobile phase B: IPA solution. A gradient (20% B at 0 min, 60% B at 5 min, 100% B at 13 min, 20% B at 13.1 to 17 min) was then initiated at a flow rate of 0.35 ml/min. MS was performed in positive and negative switch mode. Source temperature: 400 °C; electrospray ionization (ESI) positive model: ion spray voltage (IS): +3,000 V; ion source gas 1 (GS1): 50; ion source gas 2 (GS2): 55; curtain gas (CUR): 35. ESI negative model: IS: −2,500 V; GS1: 50; GS2: 55; CUR: 35. Multiple reaction monitoring (MRM) method was used for MS quantitative data acquisition. The polled quality control (QC) samples were set in the sample queue to evaluate the stability and repeatability of the system.

### Metabolomic data analysis

The semiquantitative values of glycerophospholipids obtained from MRM-based targeted analyses were first calculated by using the isotope-labeled internal standards. The normalized data matrix was autoscaled to maintain a symmetrical and comparable distribution. Multivariate statistical analysis (MVA) was established by using SIMCA-P software (v14.0, Umetrics, Umea, Sweden). Unsupervised PCA was applied to gain a comprehensive view of sample distribution and assess the outlier samples. VIP > 1.0 in the loading plots of PLS-DA and Student’s *t* test or Mann–Whitney *U* test < 0.05 represent a significant importance of the metabolic variables in differentiating groups. Chord diagram and univariate analysis were performed by using the bioinformatics platform (http://www.bioinformatics.com.cn).

### C57BL/6J WT and *LDLR^−/−^* mice treated with standard chow-containing LPA 16:0

Male C57BL/6J background *LDLR^−/−^* mice and WT mice aged 7 to 8 weeks were purchased from Beijing Huafukang Biotechnology Co. Ltd. and housed in the solid-phase extraction (SPE)-grade laboratory animal room of Beijing Anzhen Hospital of Capital Medical University, Beijing, China. The standard chow contained 10 mg/kg of LPA 16:0 or vehicle (sodium hydroxymethylcellulose). The mice were divided into 4 groups: WT + vehicle, WT + LPA 16:0, *LDLR^−/−^* + vehicle, and *LDLR^−/−^* + LPA 16:0. Each group contained 10 mice. Mice in each LPA 16:0-treated group were fed with chow-containing LPA 16:0 for 4 weeks, and mice in the vehicle-treated group were fed the corresponding vehicle chow. Sodium hydroxymethylcellulose powder and LPA 16:0 were purchased from Shanghai Yuanye Bio-technology Co. Ltd. and Cayman Chemical Co. Ltd., respectively.

### *LDLR^−/−^* mice treated with standard chow-containing palmitic acid

Male C57BL/6J background *LDLR^−/−^* mice aged 7 to 8 weeks were housed in the SPE-grade laboratory animal room. The standard chow contained 10 mg/kg of palmitic acid, 10 mg/kg of LPA 16:0, or vehicle (sodium hydroxymethylcellulose). The mice were divided into 3 groups: *LDLR^−/−^* + vehicle, *LDLR^−/−^* + LPA 16:0, and *LDLR^−/−^* + palmitic acid. Each group contained 8 mice. Mice in each group were fed for 4 weeks.

### ATX inhibitor treatment on *LDLR^−/−^* mice

Forty male C57BL/6J background *LDLR^−/−^* mice aged 7 to 8 weeks were divided into 4 groups (10 mice in each group): the chow + vehicle group, the high cholesterol diet (HCD; 1.25% cholesterol) + vehicle group, the chow + GLPG 1690 group, and the HCD + GLPG 1690 group. Mice in the 2 GLPG 1690-treated groups were gavaged with 10 mg/ml of GLPG 1690 for 4 weeks, while mice in the vehicle-treated chow and HCD groups were gavaged with 0.5% sodium hydroxymethylcellulose. Weigh 0.4 g of hydroxymethylcellulose powder dissolved in 80 ml of purified water to configure 0.5% sodium hydroxymethylcellulose solution, and place the prepared sodium hydroxymethylcellulose solution in a refrigerator at 4 °C to allow the powder to dissolve. A total of 100 mg of GLPG1690 powder (GIpBio, GC19168) was dissolved in 10 ml of 0.5% sodium hydroxymethylcellulose solution. The mixture was thoroughly mixed to prepare a homogeneous suspension with a concentration of 10 mg/ml.

### Effects of LPA 16:0 on atherosclerosis progression in *LDLR^−/−^* mice

Twenty male C57BL/6J background *LDLR^−/−^* mice aged 7 to 8 weeks were divided into 2 groups (*n* = 10), including the HCD + vehicle group and the HCD + LPA 16:0 group. Mice in the HCD + vehicle group were fed with HCD-containing 10 mg/kg LPA 16:0 for 12 weeks, and mice in the vehicle-treated group were fed with HCD-containing 10 mg/kg sodium hydroxymethylcellulose.

### Mice euthanasia, biochemical assays, and Western blot analysis

The detailed methods for mice euthanasia, sample collection, TC and TG measurements, lipoprotein distribution assays, and Western blot are depicted in the Supplementary Materials.

### Bile acid and cholesterol analyses

Briefly, 400 μl of cold methanol/acetonitrile/water (2:2:1, v/v/v) extraction solvent containing stable isotope internal standards was added to 50 mg of liver tissues or feces samples. The mixtures were under vigorous shaking for 2 min at 4 °C and incubated on ice for 20 min, and then centrifuged at 14,000*g* for 20 min at 4 °C. The supernatant was collected and flowed through a 96-well protein precipitation plate, and then the elution was collected and dried in a vacuum centrifuge at 4 °C. The dried samples were redissolved in 100 μl of acetonitrile/water (1:1, v/v) for further analysis. The quantitative analysis of primary bile acids and cholesterol in liver tissue or feces samples was performed on the Novogene Co. Ltd. QTRAP LC-MS (6500+, Sciex) platform using standard protocols.

### Transcriptomics analysis

Transcriptomics was performed on the Illumina NovaSeq 6000 (Illumina, USA) sequencing platform in Novogene Co. Ltd. (https://www.novogene.com/). Detailed methods were shown in the Supplementary Materials.

### Statistical analysis

For clinical variables, continuous data are presented as means ± SDs, and the non-normally distributed data are expressed as medians and interquartile range (IQR). Categorical variables are summarized by frequency (*N*) and percentages (%) and were compared using the chi-square test. The results of animal experiments were displayed as the mean ± SEM. Two-tailed Student’s *t* test and Mann–Whitney *U* test were used for analyzing parametric data and nonparametric data, respectively. All statistical analyses were performed with Prism version 9.0 (GraphPad Software Inc., San Diego, CA, USA). Multivariable β-coefficient regression analysis for investigating the association between the detected glycerophospholipids and the clinical conditions was performed via SPSS Statistics software (version 26, IBM Corp., New York, USA).

## Data Availability

Raw and normalized lipidomic datasets have been deposited at https://service.most.gov.cn/ and are available from the corresponding author (Y.Q.) and the manager of National Key Research and Development Program of China (J.L.) upon request following the China Human Genetic Resources Protection Law. Any additional information required to reanalyze the data reported in this paper is available from the corresponding authors on reasonable request. All requests will be evaluated within 10 working days.
