# Supplementary material for: Targeting Lysophosphatidic Acid Ameliorates Dyslipidemia in Familial Hypercholesterolemia
Source: Research (Wash D C). 2025 Feb 27;8:0629. doi: 10.34133/research.0629 (PMC11865365; doi:10.34133/research.0629)
Supplement: Supplementary 1 — Supplementary Methods Tables S1 to S3 Figs. S1 to S4 [file research.0629.f1.zip › Reporting checklist.docx]

# Reporting checklist.

Based on the STROBE cross sectional guidelines.

## Instructions to authors

Complete this checklist by entering the page numbers from your manuscript where readers will find each of the items listed below.

Your article may not currently address all the items on the checklist. Please modify your text to include the missing information. If you are certain that an item does not apply, please write "n/a" and provide a short explanation.

Upload your completed checklist as an extra file when you submit to a journal.

In your methods section, say that you used the STROBE cross sectionalreporting guidelines, and cite them as:

von Elm E, Altman DG, Egger M, Pocock SJ, Gotzsche PC, Vandenbroucke JP. The Strengthening the Reporting of Observational Studies in Epidemiology (STROBE) Statement: guidelines for reporting observational studies.

|  |  | Reporting Item | Page Number |
| --- | --- | --- | --- |
| **Title and abstract** |  | Title: Targeting lysophosphatidic acid ameliorates dyslipidemia in familial hypercholesterolemia  Short title: Targeting LPA reduces cholesterol in FH | 1 |
| Title | [#1a](https://www.goodreports.org/reporting-checklists/strobe-cross-sectional/info/#1a) | Indicate the study’s design with a commonly used term in the title or the abstract：  Herein, we aimed to comprehensively characterize the glycerophospholipid phenotypes of human FH and explore the functional roles of key glycerophospholipid features on cholesterol metabolism. Targeted analysis of 328 glycerophospholipids was used to profile the differentiated features in patients with homozygous FH (HoFH, n=181), heterozygous FH (HeFH, n=452), and non-FH hypercholesterolemia (n=382). | 1 |
| Abstract | [#1b](https://www.goodreports.org/reporting-checklists/strobe-cross-sectional/info/#1b) | Provide in the abstract an informative and balanced summary of what was done and what was found：  Our results demonstrated that the glycerophospholipid phenotypes of FH and non-FH hypercholesterolemia were dominated by a variety of metabolites in lysophosphatidic acid (LPA) metabolism. Among the differentiated LPA species, palmitoyl-LPA (16:0) showed significant association with clinical levels of LDL-C and total cholesterol in HoFH and HeFH. Using functional metabolomic strategies and murine FH model, our results indicated that supplementing with LPA 16:0 could increase the plasma levels of LDL and free/esterified cholesterol and accelerate atherosclerotic lesions in murine FH model, whereas inhibition of autotaxin-mediated LPA 16:0 production could significantly ameliorate dyslipidemia. We also mechanistically revealed a key role of LPA 16:0 on hepatic cholesterol homeostasis by impairing cholesterol excretion and bile acid synthesis. | 1 |
| **Introduction** |  |  |  |
| Background / rationale | [#2](https://www.goodreports.org/reporting-checklists/strobe-cross-sectional/info/#2) | Explain the scientific background and rationale for the investigation being reported：  Glycerophospholipids are key components of cholesterol-rich lipoproteins and the lipid bilayer of cells. An increasing body of evidence revealed that glycerophospholipid metabolism was significantly perturbed in dyslipemia. Several bioactive glycerophospholipids play as potent lipid mediators that may be involved in the interactions between genetic and environmental factors that increase susceptibility to atherosclerosis and its complications. More recently, our untargeted metabolomics study showed that several glycerophospholipid metabolites were significantly altered in the blood samples of patients with HoFH and HeFH. However, the comprehensive and precise glycerophospholipid landscapes of FH and non-FH hypercholesterolemic populations are still lacking. | 2 |
| Objectives | [#3](https://www.goodreports.org/reporting-checklists/strobe-cross-sectional/info/#3) | State specific objectives, including any prespecified hypotheses:  In the present study, we sought to systematically characterize the plasma glycerophospholipid phenotypes and features of 1,292 individuals with and without hypercholesterolemia, including HoFH, HeFH, non-FH hypercholesterolemia, and non-dyslipidemia. We also aimed to investigate the association of significantly altered glycerophospholipids with clinical cholesterol levels and explore the potential roles of key glycerophospholipid alterations in the regulation of cholesterol metabolism. | 2 |
| **Methods** |  |  | 12-15 |
| Study design | [#4](https://www.goodreports.org/reporting-checklists/strobe-cross-sectional/info/#4) | Present key elements of study design early in the paper:  A total of 1,292 individuals were enrolled in human study. Targeted profiling of 328 glycerophospholipid metabolites was performed to characterize glycerophospholipid changes in patients with homozygous FH (HoFH, n=181), heterozygous FH (HeFH, n=452), and non-FH hypercholesterolemia (n=382). Then, the biological roles of differentiated glycerophospholipid on were explored using functional metabolomic strategies and murine FH model. | 12 |
| Setting | [#5](https://www.goodreports.org/reporting-checklists/strobe-cross-sectional/info/#5) | Describe the setting, locations, and relevant dates, including periods of recruitment, exposure, follow-up, and data collection:  All FH patients were enrolled from the Familial Hypercholesterolemia Families Cohort (FHFC) affiliated to Beijing Anzhen Hospital and Peking University First Hospital between 2018 and 2023. This study complies with the Declaration of Helsinki and was approved by the Ethics Committee of Beijing Anzhen Hospital of the Capital University of Medical Sciences and Peking University First Hospital. Ethics approval was obtained from the Ethics Committee of Beijing Anzhen Hospital of the Capital University of Medical Sciences (2017035) and Peking University First Hospital (2022143-005). Verbal and written consent was obtained from all subjects. | 12 |
| Eligibility criteria | [#6](https://www.goodreports.org/reporting-checklists/strobe-cross-sectional/info/#6a) | Give the eligibility criteria, and the sources and methods of selection of participants:  All subjects that meet the inclusion criteria will be included. Each group contained over 100 study individuals. | 12 |
|  | [#7](https://www.goodreports.org/reporting-checklists/strobe-cross-sectional/info/#7) | Clearly define all outcomes, exposures, predictors, potential confounders, and effect modifiers. Give diagnostic criteria, if applicable:  All FH patients were genetically confirmed with two (HoFH) or one mutant (HeFH) alleles at the genes encoding of LDLR. Non-FH was defined as subjects with lower levels of untreated LDL-C than 4.7 mmol/L and without FH-related mutations in gene encoding LDLR, apolipoprotein B, proprotein convertase subtilisin/kexin type 9, or LDLR adaptor protein 1. ASCVD history for the subjects at inclusion were defined as a composite of myocardial infarction, coronary and carotid revascularization, ischemic or atherothrombotic stroke. Non-FH hypercholesterolemia and non-dyslipidemia individuals were enrolled from Beijing Anzhen Hospital between 2018 and 2022. Non-FH hypercholesterolemia was defined as patients with LDL-C levels ranging from 3.65 to 4.7 mmol/L and without FH-related gene mutations in genetic sequencing. Non-dyslipidemia was defined as individuals without clinical diagnosis of hypercholesterolemia, hyperphytosterolemia, and hypertriglyceridemia or history of lipid-lowering medications. Exclusion criteria for all the study subjects: current or historical diseases or conditions of respiratory diseases, serious digestive diseases, infectious diseases, chronic kidney diseases, pregnancy, and malignancy. | 13 |
| Quantitative variables | [#8](https://www.goodreports.org/reporting-checklists/strobe-cross-sectional/info/#7) | Explain how quantitative variables were handled in the analyses. If applicable, describe which groupings were chosen, and why:  For human study, a total of 100 µL plasma was firstly mixed with 200 µL methanol containing 10 µL internal deuterated-phospholipid standards (LPA 16:0-*d*_9_, PA 15:0/18:1-*d*_7_, LPC 14:0-*d*_7_, PC 16:0-*d*_3_, LPE 16:0 -*d*_9_, PE 17:0/18:1-*d*_5_, PC 16:0/18:2-*d*_5_, LPI 19:0-*d*_5_, PI 17:0/16:1-*d*_5_, LPS 19:0-*d*_5_, PS 17:0/18:1-*d*_5_, LPG 19:0-*d*_5_, PG 17:0-16:1-*d*_5_), then 800 µL MTBE were added. The mixture was adequately vortexed, sonicated for 30 min at 4℃ and then kept for 20 min. After that, 200 µL of LC-MS-grade deionized water was added, and the mixture was vortexed and centrifuged at 14000 rpm for 15min at 4℃. The upper organic solvent layer was obtained and dried under nitrogen. Then, the samples were re-dissolved in 200 µL of IPA/CAN (9:1, v/v) for further LC-MS analysis. For animal study, a total of 100 µL plasma and 50mg liver were used, and the glycerophospholipids extraction was the same as that in human study.  The LC/MS analysis was performed on a UHPLC system (LC-30AD, Shimadzu) coupled with QTRAP MS (6500+, Sciex) platform in Novogene Co., Ltd. The analytes were separated on C18 column (Phenomenex, Kinetex C18, 2.1x100 mm, 2.6 μm). Column temperature was set at 45℃. Mobile phase A: 70% acetonitrile+30% H_2_O + 5mM ammonium acetate, mobile phase B: IPA solution. A gradient (20% B at 0 min, 60% B at 5 min, 100% B at 13 min, 20% B at 13.1-17 min) was then initiated at a flow rate of 0.35 mL/min. MS was performed in positive and negative switch mode. Source temperature: 400℃; ESI positive model: Ion Spray Voltage (IS): +3000 V; Ion Source Gas1 (GS1): 50; Ion Source Gas2 (GS2): 55; Curtain Gas (CUR): 35. ESI negative model: IS: -2500 V; GS1: 50; GS2: 55; CUR: 35. MRM method was used for mass spectrometry quantitative data acquisition. A polled quality control (QC) samples were set in the sample queue to evaluate the stability and repeatability of the system. | 13 |
| Statistical method 1 | [#9a](https://www.goodreports.org/reporting-checklists/strobe-cross-sectional/info/#12a) | Describe all statistical methods, including those used to control for confounding:  The semi-quantitative values of glycerophospholipids obtained from MRM-based targeted analyses were firstly calculated by using the isotope-labeled internal standards. The normalized data matrix was auto-scaled to maintain a symmetrical and comparable distribution. Multivariate statistical analysis (MVA) was established by using SIMCA-P software (v14.0, Umetrics, Umea, Sweden). Unsupervised PCA was applied to gain a comprehensive view of samples distribution and assess the outlier samples. VIP > 1.0 in the loading plots of PLS-DA, Student’s t test or Mann Whitney U test <0.05 represent a significant importance of the metabolic variables in differentiating groups. Chord diagram and univariate analysis was performed by using bioinformatics platform (http://www.bioinformatics. com.cn). | 13-14 |
| Statistical method 2 | [#9b](https://www.goodreports.org/reporting-checklists/strobe-cross-sectional/info/#12b) | Describe any methods used to examine subgroups and interactions:  For clinical variables, continuous data are presented as the means and standard deviations (means ± SDs), and the non-normally distributed data are expressed as medians and interquartile range [IQR]. Categorical variables are summarized by frequency (N) and percentages (%) and were compared using the chi-square test.The results of animal experiments were displayed as the mean ± SEM. Two-tailed Student’s t test and Mann-Whitney U test was used for analyzing parametric data and nonparametric data, respectively. All statistical analyses were performed with Prism version 9.0 (GraphPad Software, Inc., San Diego, CA, USA). | 15 |
| **Results** |  |  |  |
| Participants | [#10](https://www.goodreports.org/reporting-checklists/strobe-cross-sectional/info/#13a) | Report numbers of individuals at each stage of study—eg numbers potentially eligible, examined for eligibility, confirmed eligible, included in the study, completing follow-up, and analysed. Give information separately for for exposed and unexposed groups if applicable:  Give characteristics of study participants (eg demographic, clinical, social) and information on exposures and potential confounders. Give information separately for exposed and unexposed groups if applicable.  A total of 633 FH patients with genetically confirmed LDLR mutations from two independent FH centers (Beijing Anzhen Hospital and Peking University First Hospital) participated in this study, containing 181 HoFH subjects (male, 52.5%) and 452 HeFH patients (male, 51.3%). Another 112 non-FH individuals (male, 55.4%) were enrolled as a comparison group. Clinical characteristics are presented in Table S1. Patients with HoFH had highest levels of LDL-C (14.12±5.36 mmol/L) compared with HeFH (5.31±1.37 mmol/L) and non-FH (2.21±0.43 mmol/L) individuals. Besides, historical ASCVD was recorded in thirty-two subjects with HoFH (17.6%). In the non-FH hypercholesterolemic set, a total of 382 patients (male, 51.1%) were enrolled from Beijing Anzhen Hospital (Table S2). Compared to non-dyslipidemia individuals (n=165; male, 54.6%), non-FH hypercholesterolemic patients showed higher levels of LDL-C and total cholesterol (TC). No significant difference was observed in the prevalences of hypertension and diabetes mellitus in each subgroup comparison. | 2-3 |
| Main results | [#11](https://www.goodreports.org/reporting-checklists/strobe-cross-sectional/info/#16a) | Give unadjusted estimates and, if applicable, confounder-adjusted estimates and their precision (eg, 95% confidence interval). Make clear which confounders were adjusted for and why they were included:  Considering that lipid-lowering therapy (LLT) are potential factors that might affect the plasma lipids, we also employed unsupervised PCA analysis to explore the potential effects of LLT on the glycerophospholipid profiles from different study groups. As shown in the **Figure S1C** and **D**, the glycerophospholipid profiles did not show clustering of samples by LLT status in the PCA score plots of FH and non-FH hypercholesterolemia populations, indicating no discriminatory features due to the differences in LLT status. Furthermore, we also performed multivariate regression analyses to assess the integrated effects of several important metabolic factors (including LLT, age, sex, ASCVD history, hypertension, and type 2 diabetes mellitus) on the differentiated glycerophospholipid species. As shown in the **Figure 3**, our results demonstrated that most of the identified lysophospholipid markers (LPA and LPC species) in the multivariate and univariate statistical analyses were still significantly associated with HoFH, HeFH, or non-FH hypercholesterolemia conditions (*p* values < 0.05). These results indicated that the commonly known metabolic factors showed few effects on the characteristic glycerophospholipid profiles in patients with FH or non-FH hypercholesterolemia. | 4 |
| **Discussion** |  |  |  |
| Key results | [#12](https://www.goodreports.org/reporting-checklists/strobe-cross-sectional/info/#18) | Summarise key results with reference to study objectives:  To our knowledge, this is the first study to characterize the glycerophospholipid metabolism phenotypes of patients with FH and non-FH hypercholesterolemia. The main findings of our study are the following: First, patients with FH and non-FH hypercholesterolemia exhibited profound alterations in a variety of lysophospholipids. Second, LPA species, in piratically LPA 16:0, significantly correlated with the plasma cholesterol levels of patients with HoFH and HeFH. Third, this study demonstrated that inhibition of ATX–mediated LPA 16:0 production could significantly reduce the circulating cholesterol levels by regulating hepatic cholesterol excretion and primary bile acid synthesis in genetic mice model that mimic human HoFH, providing potential intervention targets for improving severe hypercholesterolemia in HoFH population. | 11 |
| Limitations | [#13](https://www.goodreports.org/reporting-checklists/strobe-cross-sectional/info/#19) | Discuss limitations of the study, taking into account sources of potential bias or imprecision. Discuss both direction and magnitude of any potential bias.  Further studies on exploring the effects of liver-specific ATX inhibition and ATX inhibitors on atherosclerosis progression in LDLR–/– mice are warranted. The long-term effects and safety assessments of ATX inhibition may be worthwhile. Moreover, further studies designed to investigate the genetic associations of ATX and clinical phenotypes in FH population may offer great promise for exploring additional intervention strategies to lower cholesterol and combat atherosclerosis progression. | 12 |
| Funding | [#14](https://www.goodreports.org/reporting-checklists/strobe-cross-sectional/info/#22) | Give the source of funding and the role of the funders for the present study and, if applicable, for the original study on which the present article is based:  This work was supported by the Noncommunicable Chronic Diseases-National Science and Technology Major Project (Nos. 2023ZD0503400 and 2023ZD0503403), the National Key Research and Development Program of China (Grant No. 2021YFC2500603) and the National Natural Science Foundation of China (Grant Nos. 82100295). | 15 |
| **Other Information** | [#15](https://www.goodreports.org/reporting-checklists/strobe-cross-sectional/info/#22) |  |  |

None The STROBE checklist is distributed under the terms of the Creative Commons Attribution License CC-BY. This checklist can be completed online using <https://www.goodreports.org/>, a tool made by the [EQUATOR Network](https://www.equator-network.org) in collaboration with [Penelope.ai](https://www.penelope.ai)
