# Supplementary material for: Targeting Lysophosphatidic Acid Ameliorates Dyslipidemia in Familial Hypercholesterolemia
Source: Research (Wash D C). 2025 Feb 27;8:0629. doi: 10.34133/research.0629 (PMC11865365; doi:10.34133/research.0629)
Supplement: Supplementary 1 — Supplementary Methods Tables S1 to S3 Figs. S1 to S4 [file research.0629.f1.zip › Revised-Supplemental Material.docx]

***Supplemental Material***

**Supplementary methods**

**Mice euthanasia**

Mice were fasted for 4-6 hours before sampling, and mice were injected intraperitoneally with 1% pentobarbital solution, weighed, and euthanized. The abdominal and thoracic cavities were quickly cut open, and the heart was exposed for apical blood sampling, which was performed with a 1mL syringe containing EDTA, and the removed blood was injected into a 1.5 mL EP tube, gently mixed to prevent coagulation; the right auricle was cut open, and the blood was allowed to drain out by instilling 20 mL of PBS solution from the left ventricle; the livers were quickly collected, and the livers of the mice were weighed; liver tissues of the mice and the small intestine tissues of the mice were put into a 1.5 mL EP tube. The blood in the EP tubes was then centrifuged at 3000 rpm for 15 minutes at 4℃. After centrifugation, the upper layer of plasma was transferred to new EP tubes and put into a -80℃ refrigerator for long-term storage for subsequent experiments. The remaining liver tissue and other organ tissues were immediately snap-frozen in liquid nitrogen. The tissues were retrieved from liquid nitrogen and transferred to a -80℃ refrigerator for storage for subsequent experiments. The mouse aorta in the HCD + vehicle group, the HCD + LPA 16:0 group was stripped under a dissecting microscope, and the adipose and connective tissues adjacent to the aorta were carefully stripped; the mouse aorta was placed into an EP tube. The aorta was fixed in 4% paraformaldehyde solution for subsequent pathological sectioning.

**Plasma TG and TC were detected by end-point colorimetric assay**

Plasma TC and TG levels were measured by using the Biosino Bio-technology and Science Co. Kit, and the method according to the instruction of the kit is as follows: The working solution for TG and TC was prepared by adding 10 mL of R2 solution to R1 solution, mixing it well and preparing it for use. Add 5 μL of plasma and 200 μL of working solution to the plate and incubate at 37℃ for 10 minutes. Incubate the plate with 5 μL of plasma and 200 μL of working solution at 37 ℃ for 10 minutes, detect the absorbance at 492 nm, and obtain the standard curve and sample concentration according to the data of the standard.

**Plasma lipoprotein distribution assay**

Plasma lipoprotein levels were analyzed by running 100 μL pooled serum onto a gel-filtration FPLC system. Samples were loaded on a GE Superose 6 10/30 GL column in 0.15 M sodium chloride containing 1 mM EDTA and 0.02% sodium azide, pH 7.4. Fractions (0.5 mL) were collected (0.5 mL/min). A lipid extraction kit (Biovision) was used for hepatic lipid extraction. The levels of total cholesterol in each fraction were determined by using cholesterol assay kits (SEKISUI Diagnostics).

**Targeted analyses of bile acids, cholesterol, and cholesterol ester**

For lipid metabolites extraction, 400 μL of cold methanol/acetonitrile/water (2:2:1, v/v/v) extraction solvent containing stable-isotope internal standards was added to 50 mg liver tissues, 50mg feces, or 100 μL plasma samples. The mixtures were under vigorous shaking for 2 min at 4°C and incubated on ice for 20 minutes, and then centrifuged at 14,000 g for 20 minutes at 4°C, the supernatant was collected and flowed through a 96-well protein precipitation plate, and then the elution was collected and dried in a vacuum centrifuge at 4°C. The dried samples were re-dissolved in 100 μL acetonitrile/water (1:1, v/v) for further analysis. Using standard protocols, the quantitative analysis of primary bile acids and free cholesterol in liver tissues or feces samples were performed on the Novogene Co., Ltd. QTRAP LC-MS (6500+, Sciex) platform. The free cholesterol and cholesterol ester (CE) in the plasma samples were performed MetWare (http://www.metware.cn/) following standard protocols.

**Western blot analysis**

The protein samples were extracted from the lysate of mice liver tissues and Huh7 cells. Twenty-milligram samples of liver tissue were lysed in RIPA buffer (Solarbio, Beijing, China) by ultrasonication, the lysates were centrifuged at 12,000 rpm for 20 min, the supernatants were collected, and the protein content was analyzed using western blot analysis. Proteins were separated on sodium dodecyl sulfate-polyacrylamide gel electrophoresis and then transferred onto PVDF membranes. The membranes were blocked using 5% skimmed milk and then sequentially incubated with appropriate primary and secondary antibodies. Western blot analysis was performed using with the following antibodies: anti-LIPA (Proteintech, catalog 12956-1-AP), anti-ABCG8 (Proteintech, catalog 24453-1-AP), anti-CYP39A1 (Thermofisher, catalog PA5-101317) anti-CYP27A1 (Proteintech, catalog 67045-1-Ig), anti-CPY7A1(Proteintech, catalog 18054-1-AP).

**Transcriptomics analysis**

Transcriptomics was performed on the Illumina NovaSeq 6000 (Illumina, USA) sequencing platform in Novogene Co., Ltd. (https://www.novogene.com/). Briefly, total RNA was extracted from liver tissues. Using fragmented mRNA as a template and random oligonucleotides as primers, the first strand of cDNA was synthesized under the action of M-MuLV reverse transcriptase. Then, RNaseH was used to degrade the RNA strand, and the first strand of the cDNA was synthesized with dNTPs under the DNA polymerase I system. The second strand of cDNA is then synthesized using dNTPs as raw material under the DNA polymerase I system. After synthesis, the double-stranded cDNA was purified, end-repaired, and connected to the sequencing connector. Next, about 250 cDNAs were screened using AMPure XP beads was used to screen out cDNA fragments of about 250-300bp size for PCR amplification. After amplification, the PCR products were purified again using AMPure XP beads to obtain the desired library. Then the different libraries were inspected and pooled according to their effective concentration and the target downstream data volume. Subsequently, the Illumina sequencing platform was used to perform high-throughput sequencing on these mixed libraries. The sequencing platform of Illumina was then used to perform high-throughput sequencing of these mixed libraries. The differentiated mRNAs were identified by volcano plot.

**Table S1. Demographic and clinical characteristics of HoFH, HeFH, and non-FH subjects.**

|  | HoFH  (n=181) | HeFH  (n=452) | Non-FH  (n=112) | *p* value |
| --- | --- | --- | --- | --- |
| Age, years | 22.4±12.6 | 38.3±13.6 ^a^ | 23.2±12.4 | <0.0001 |
| Male sex, n (%) | 95 (52.5%) | 232(51.3%) | 62(55.4%) | 0.74 |
| Hypertension, n (%) | 4 (2.2%) | 16 (3.5%) | 4 (3.6%) | 0.68 |
| Type 2 diabetes mellitus, n (%) | 1 (0.6%) | 5 (1.1%) | 2 (1.8%) | 0.61 |
| ASCVD history, n (%) | 32 (17.7%) | 14 (3.1%) | 0 (0.0%) | <0.0001 |
| LDL-C, mmol/L | 14.12±5.36 | 5.31±1.37 | 2.21±0.43 | <0.0001 |
| TC, mmol/L | 16.24±5.82 | 7.26±1.74 | 4.12±0.48 | <0.0001 |
| TG, mmol/L | 1.11 [0.72, 1.41] | 1.31 [0.82, 1.75] | 0.73 [0.54, 0.86] | 0.0021 |
| HDL-C, mmol/L | 0.94±0.33 | 1.39±0.52 | 1.51±0.32 | <0.0001 |
| Lipid-lowering therapy, n (%) | 158 (87.3%) | 266 (58.8%) | 34 (30.4%) | <0.0001 |

Continuous data are presented as mean ± standard deviation or median [interquartile range], and categorical variables are presented as %. One-ANOVA were used for continuous data. The Chi-square test was used for categorical data. Abbreviations: atherosclerotic cardiovascular disease, ASCVD; low-density lipoprotein cholesterol, LDL-C; high-density lipoprotein cholesterol, HDL-C; total cholesterol, TC; triglyceride, TG; heterozygous familial hypercholesterolemia, HeFH; homozygous familial hypercholesterolemia, HoFH.

**Table S2. Demographic and clinical characteristics of non-FH hypercholesterolemia and non-dyslipidemia individuals.**

| ID | non-FH hypercholesterolemia (n=382) | Non-dyslipemia (n=165) | *p* value |
| --- | --- | --- | --- |
| Age, years | 42.2±14.5 | 45.6±15.2 | 0.64 |
| Male sex, n (%) | 195(51.1%) | 90(54.6%) | 0.45 |
| Hypertension, n (%) | 145 (38.0%) | 51 (30.9%) | 0.11 |
| Type 2 diabetes mellitus, n (%) | 24 (6.3%) | 6 (3.6%) | 0.21 |
| ASCVD history, n (%) | 13 (3.4%) | 1 (0.6%) | 0.057 |
| LDL-C, mmol/L | 3.95±0.62 | 2.39±0.51 | <0.0001 |
| TC, mmol/L | 5.26±0.87 | 3.76±0.48 | <0.0001 |
| TG, mmol/L | 0.84 [0.71, 1.18] | 0.78 [0.61, 1.22] | 0.43 |
| HDL-C, mmol/L | 1.14±0.29 | 1.21±0.32 | 0.51 |
| Lipid-lowering therapy, n (%) | 83 (21.7%) | 0 (0.0. %) | <0.0001 |

Continuous data are presented as mean ± standard deviation or median [interquartile range], and categorical variables are presented as %. Two-tailed Student’s *t* test or Mann Whitney *U* test were used for continuous data. The Chi-square test was used for categorical data. Abbreviations: atherosclerotic cardiovascular disease, ASCVD; low-density lipoprotein cholesterol, LDL-C; high-density lipoprotein cholesterol, HDL-C; total cholesterol, TC; triglyceride, TG.

**Table S3. The values of coefficient of variation and relative standard deviation for all deuterated standard substances in the quality control samples.**

| Metabolomic analysis in FH and non-FH subjects | | | |
| --- | --- | --- | --- |
| ID | Coefficient of variation | Relative standard deviation for retention times (%) | Relative standard deviation for peak areas (%) |
| LPA 16:0-*d9* | 0.13 | 0.28 | 3.14 |
| PA 15:0/18:1-*d7* | 0.15 | 0.43 | 3.65 |
| LPC 14:0-*d7* | 0.11 | 0.37 | 3.84 |
| PC 16:0-*d3* | 0.18 | 0.43 | 2.31 |
| LPE 16:0-*d9* | 0.17 | 0.26 | 2.98 |
| PE 17:0/18:1-*d5* | 0.22 | 0.37 | 3.61 |
| PC 16:0/18:2-*d5* | 0.17 | 0.35 | 3.58 |
| LPI 19:0-*d5* | 0.21 | 0.29 | 3.21 |
| PI 17:0/16:1-*d5* | 0.17 | 0.34 | 2.89 |
| LPS 19:0-*d5* | 0.16 | 0.26 | 2.23 |
| PS 17:0/18:1-*d5* | 0.19 | 0.32 | 2.82 |
| LPG 19:0-*d5* | 0.18 | 0.36 | 3.47 |
| PG 17:0-16:1-*d5* | 0.16 | 0.29 | 3.45 |
| Metabolomic analysis in non-FH hypercholesterolemia and non-dyslipemia individuals | | | |
| ID | Coefficient of variation | Relative standard deviation for retention times (%) | Relative standard deviation for peak areas (%) |
| LPA 16:0-*d9* | 0.12 | 0.36 | 2.65 |
| PA 15:0/18:1-*d7* | 0.13 | 0.41 | 3.12 |
| LPC 14:0-*d7* | 0.094 | 0.42 | 3.41 |
| PC 16:0-*d3* | 0.15 | 0.41 | 1.95 |
| LPE 16:0-*d9* | 0.13 | 0.23 | 2.67 |
| PE 17:0/18:1-*d5* | 0.21 | 0.34 | 3.41 |
| PC 16:0/18:2-*d5* | 0.16 | 0.41 | 3.47 |
| LPI 19:0-*d5* | 0.19 | 0.26 | 2.98 |
| PI 17:0/16:1-*d5* | 0.18 | 0.32 | 2.62 |
| LPS 19:0-*d5* | 0.14 | 0.16 | 1.88 |
| PS 17:0/18:1-*d5* | 0.17 | 0.31 | 2.64 |
| LPG 19:0-*d5* | 0.14 | 0.31 | 3.21 |
| PG 17:0-16:1-*d5* | 0.15 | 0.27 | 3.12 |

Abbreviations: phosphatidylcholine, PC, lysophosphatidylcholine, LPC; phosphatidic acid, PA; lysophosphatidic acid, LPA; phosphatidylethanolamine, PE, lysophosphatidylethanolamine, LPE; phosphatidylglycerol, PG; lysophosphatidylglycerol, LPG; phosphatidylserine, PS; lysophosphatidylserine, LPS; phosphatidylinositol, PI; lysophosphatidylinositol, LPI.


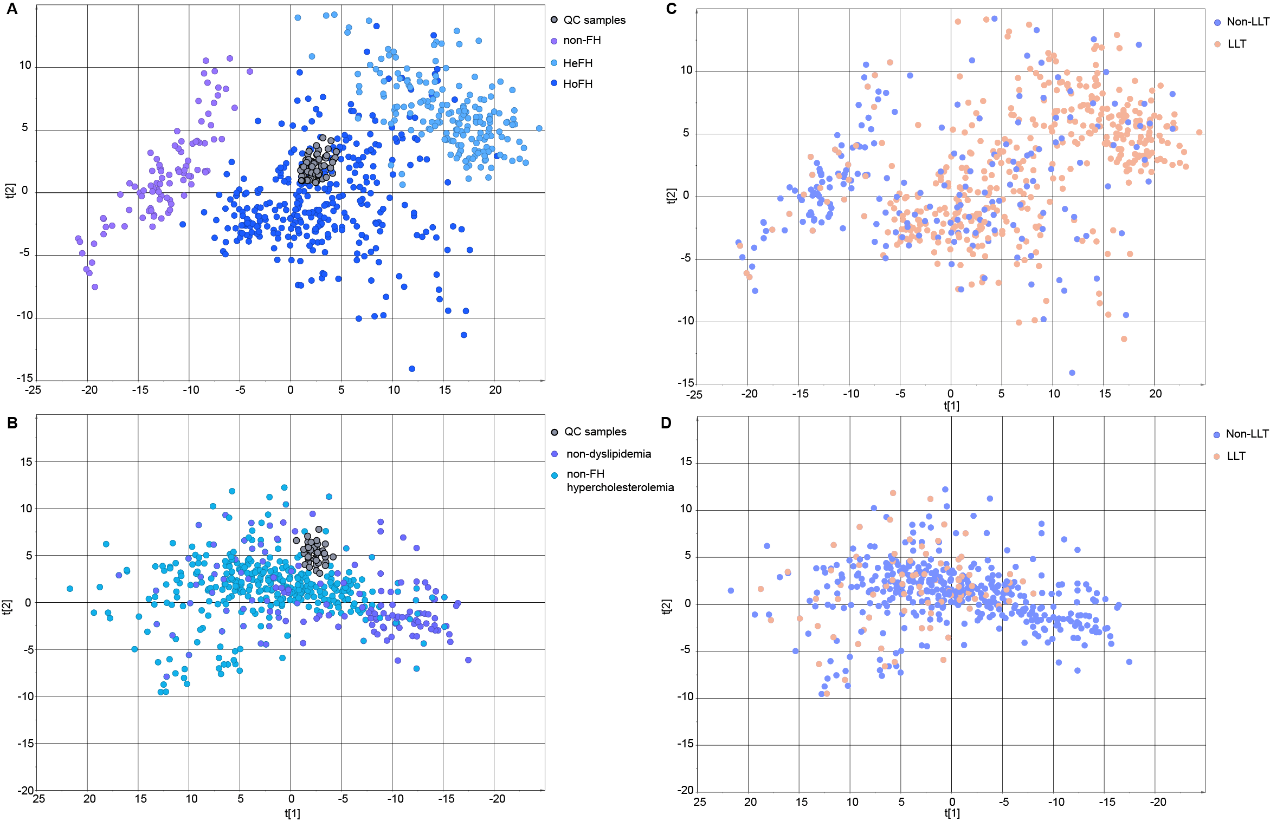


**Figure S1. Stability assessment of the metabolomic data from FH and non-FH hypercholesterolemia population. (A)** Principal component analysis (PCA) scores plot of all quality control (QC) samples and test samples (HoFH, HeFH, and non-FH individuals). **(B)** PCA scores plot of all QC samples and test samples (non-FH hypercholesterolemia and non-dyslipemia individuals). **(C)** PCA score plot of all detected glycerophospholipids from FH and non-FH individuals showing no variance due to lipid-lowering therapy (LLT). **(D)** PCA score plot of all detected glycerophospholipids from non-FH hypercholesterolemia and non-dyslipemia individuals showing no variance due to LLT. Abbreviations: heterozygous familial hypercholesterolemia, HeFH; homozygous familial hypercholesterolemia, HoFH.


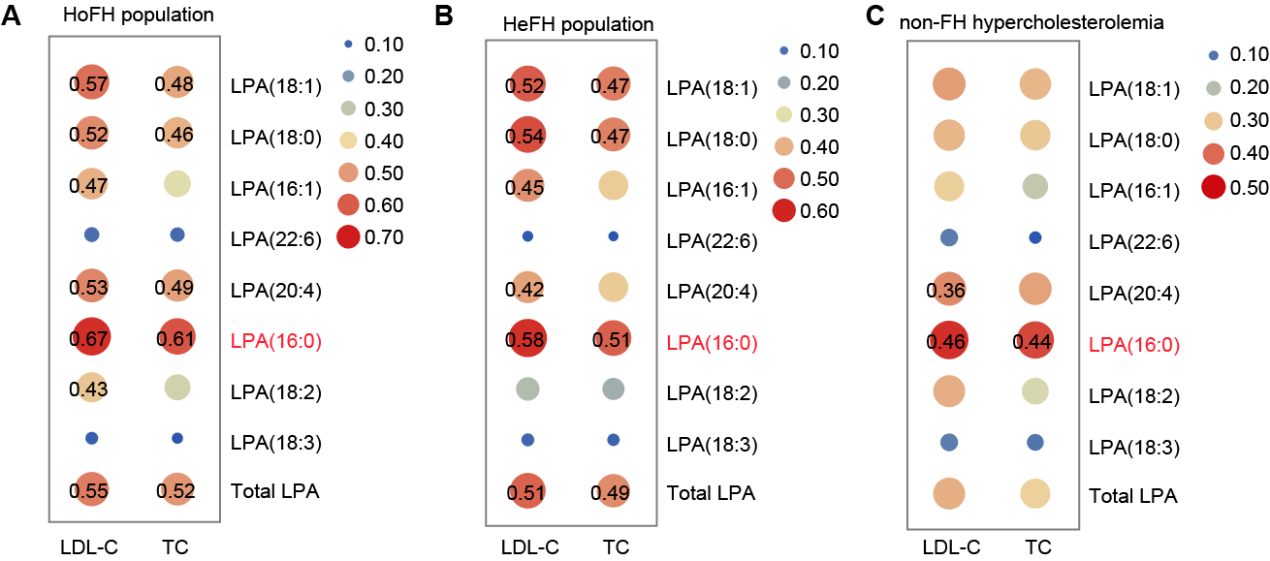


**Figure S2. The associations of lysophosphatidic acid (LPA) with clinical cholesterol levels.** **(A)** Correlations of individual LPA and total LPAs with the plasma levels of LDL-C and TC in HoFH population using Spearman correlation analysis. **(B)** Correlations of individual LPA and total LPAs with the plasma levels of LDL-C and TC in HeFH population. **(C)** Correlations of individual LPA and total LPAs with the plasma levels of LDL-C and TC in non-FH hypercholesterolemia population. Only coefficient with a statistically significant *p* value < 0.05 is labeled in the plot. Abbreviations: low-density lipoprotein cholesterol, LDL-C; total cholesterol, TC.


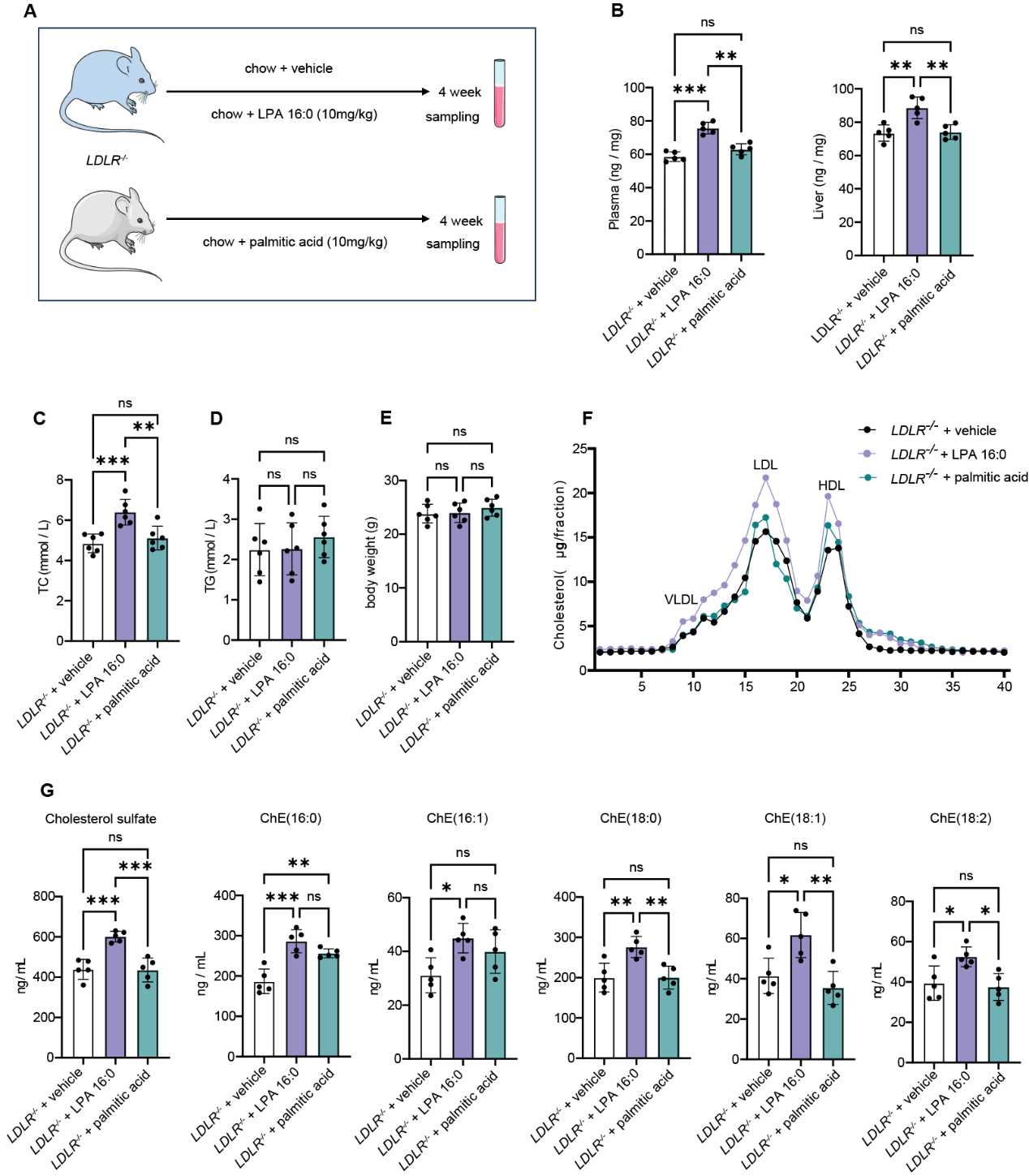


**Figure S3.** **Effects of LPA 16:0 and palmitic acid on the plasma lipid levels of *LDLR^–/–^* mice.** **(A)** Study design for investigating the effects of chow-containing LPA 16:0 and palmitic acid in *LDLR^–/–^*mice. **(B)** The plasma and liver levels of LPA 16:0 after four consecutive weeks of chow supplementation. **(C)** Plasma levels of total cholesterol (TC) in different groups. **(D)** Plasma levels of triglyceride (TG) in different groups. **(E)** The values of body weight in different groups. **(F)** Cholesterol content in VLDL, LDL, and HDL fractioned by fast protein liquid chromatography. **(G)** The fecal levels of free cholesterol sulfate and cholesterol ester (ChE). Data are expressed as means ± SEM, *, **, or *** indicated *p* < 0.05, *p* < 0.01, or *p* < 0.001 respectively. ns, no statistical differences. Abbreviations: very low-density lipoprotein, VLDL; low-density lipoprotein, LDL; high-density lipoprotein, HDL.


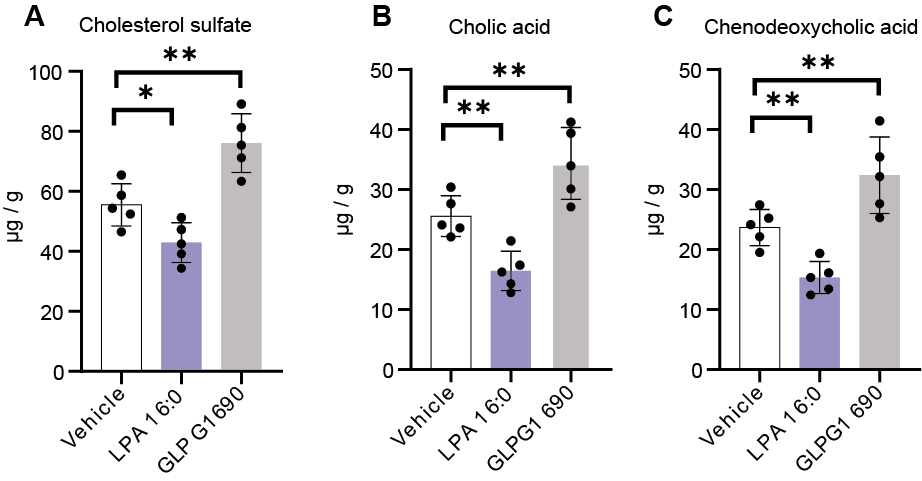


**Figure S4. The fecal levels of free cholesterol sulfate and primary bile acids between different groups. (A)** Free cholesterol sulfate. **(B)** Cholic acid. **(C)** Chenodeoxycholic acid. ** or * indicated *p* < 0.01 or *p* < 0.05.
